# Modulation of cyclophosphamide-induced immunosuppression and intestinal flora in broiler by deep eutectic solvent extracted polysaccharides of *Acanthopanax senticosus*

**DOI:** 10.3389/fvets.2024.1415716

**Published:** 2024-05-27

**Authors:** Jianqing Su, Jiaojiao Xue, Xueyan Wang, Rui Zhang, Xueping Zhang, Yaosen Yang, Xiuling Chu

**Affiliations:** College of Agronomy and Agricultural Engineering, Liaocheng University, Liaocheng, China

**Keywords:** *Acanthopanax senticosus* polysaccharide, cyclophosphamide, gut microbiota, immune suppression, 16S rRNA

## Abstract

**Introduction:**

The aim of this experiment was to investigate the modulation effect of *Acanthopanax senticosus* polysaccharide (ASPS-PD) extracted with deep eutectic solvent on cyclophosphamide-induced immunosuppression in broilers and its modulation of the gut microbiota of broilers.

**Methods:**

The 108 one-day-old broilers were divided into six groups, including the control group, the Cyclophosphamide (CY) model group, the ASPS-PD control group, the ASPA-PD high and low dose groups and the *Astragalus* polysaccharide group. Body weight, feed intake, feed conversion ratio, and immune organ index of broilers at 7, 14, and 21 days were determined; IL-2, IFN-γ, and lgG1 levels were determined by enzyme-linked immunosorbent assay (ELISA); Broiler caeca feces were analyzed by amplification and 16S rRNA sequencing.

**Results:**

The results showed that ASPS-PD can restore growth performance, increase immune organ index and improve serum cytokine levels of IL-2 and IFN-γ and immunoglobulin lgG1 levels in CY-treated broilers. The analysis of cecum flora showed that ASPS-PD can promote the proliferation of beneficial bacteria and reduce the number of harmful bacteria, regulating intestinal flora.

**Discussion:**

Therefore, ASPA-PD may be a potential novel immunomodulator to ameliorate CY-induced immunosuppression and intestinal flora dysregulation in broiler.

## 1 Introduction

Impaired immunity is a transient or chronic disruption of the immune system caused by a variety of factors, including the environment, toxins, and chemicals, with infectious diseases having a significant impact on the poultry industry. Infectious Bursal Disease, Salmonella, Avian Influenza, Newcastle Disease, and mixed infections of several diseases continue to plague the poultry industry (1–5). These immunosuppressive viruses increase broilers' morbidity and mortality and cause farmers significant economic losses. Cyclophosphamide is considered an immunosuppressive compound, and it has been reported that administration of CY to newly hatched chickens resulted mainly in B-cell damage and the development of humoral immunosuppression (6–10). Therefore, CY has been used to study the immunomodulatory effects of plant extracts (11). In addition, CY disrupts the intestinal mucosa and the composition of the microorganisms in the gut (12, 13). Therefore, the broiler model of CY immunosuppression was chosen for this experiment.

Polysaccharides have attracted attention for their therapeutic effects. They are considered immune molecules of the innate immune system with less toxicity and side effects (14, 15). Studies have shown that plant-derived polysaccharides have a variety of biological activities, the most notable of which are immunomodulatory effects. Polysaccharides also have antiviral and anti-inflammatory properties (14, 16, 17). *Acanthopanax senticosus* (AS) belongs to the genus *Acanthopanax* in the family *Wujiaceae*, and the whole plant can be used as a medicine with potent antioxidant and immunomodulatory pharmacological activities. The main components are flavonoids, polyphenols, polysaccharides, etc. Yang et al. found that polysaccharides from AS could promote the proliferation of lymphocytes and improve the vaccination of regular and immunosuppressed chickens (18). Kong et al. found that AS extracts as feed additives could enhance weaned piglets' cellular and humoral immune responses by modulating the production of immune cells, cytokines, and antibodies (19). Chen et al. found that soluble polysaccharides extracted from the leaves of AS can exhibit strong immunomodulatory activity against lymphocyte proliferation *in vitro* (20). Han et al. showed that AS polysaccharides regulate immunity by activating B cells and macrophages through TLR signaling (21). Therefore, AS polysaccharides have some research potential as safe green feed additives to enhance immunity in poultry.

The intestinal tract is not only the main immune organ but also a vital digestive organ with a barrier function. It can prevent various parasitic microorganisms and their secreted toxins from spreading to extra-intestinal tissues and organs and effectively prevent endogenous microorganisms and toxins from harming the animal. It provides nutrients and a survival environment for intestinal micro-organisms, which play an important part in sustaining gut health, nutrition and the body's immunity (22, 23). Once this mutually advantageous co-regulation is broken, the intestinal immune system is disrupted and dysbiosis of the bacterial microbiota can lead to reduced immune function (24). Maintaining gut health is, therefore, a critical task. In addition, polysaccharides have been shown to enhance the body's immunity by modulating the gut flora. Polysaccharides extracted from *Ginseng, Cordyceps sinensis* and *Lycopod barbarum* (25–27) plants, for that matter, can modulate the immune response by improving the enteromucosal boundary and by promoting the multiplication of favorable microorganisms that produce short-chain fatty acids. Long et al. have shown that dietary supplementation with AS polysaccharides can improve the number of lactic acid bacteria in the broiler intestine (28). The dietary addition of AS extract dramatically enhanced the numbers of *Lactobacillus* and *Bacillus subtilis* and lowered the populations of *Escherichia coli* and *Salmonella* in the ileum (29).

The modulatory effects of ASPS-PD on immunity and gut microbiota in immunosuppressed broilers have not previously been investigated. Therefore, the present study aimed to assess the modulatory activities of ASPS-PD on the immunosuppressed broiler model of cyclophosphamide and gut microbiota and to provide strong evidence for the use of ASPS-PD as a safe and environmentally friendly feed additive to enhance the immunity of poultry organisms.

## 2 Materials and methods

### 2.1 Reagents and materials

AS were purchased from Liaocheng Limin Pharmacy and ground into powder using a pulveriser (JYZ-B521, Joyoung Co., Ltd.). L-malic acid, L-proline, and Cyclophosphamide were purchased from Shanghai Macklin Biochemistry & Technology Co., Ltd. The kits for IL-2, IFN-γ, and IgG1 were purchased from Jiangsu Enzyme Immunity Industry Co. Ltd.

### 2.2 Extraction of ASPS-PD from AS

Based on the results of previous studies, ASPS-PD were prepared using a deep eutectic method under optimal extraction conditions (30). Malic acid: proline = 1: 4 was used as the extraction solvent, and the extraction of ASPS-PD was carried out under the conditions of ultrasonic power of 240 W, material-liquid ratio of 31 g/mL, water content of 32%, extraction time of 129 min and extraction temperature of 60°C. The extract was centrifuged, and the supernatant was added anhydrous ethanol to at a final concentration of 80% and preserved at 4°C for 24 h. The mixtures was centrifuged, the residue was dried at 60°C and then ground to powder to obtain the ASPS-PD.

### 2.3 Animals experimental design

A total of 108 broilers were used in this experiment, all experimental animals were purchased from Shandong Experimental Animal Center, animal experiments were approved by Institutional Animal Care and Use Committee of Liaocheng University (protocol code: 2023022732), all procedures were carried out in accordance with the Basel Declaration and the relevant policies of China.

One-day-old broilers (42.77 ± 3.11 g) were selected and acclimatized before the start of the experiment. The broilers were kept under appropriate conditions of ventilation, light, relative humidity and temperature, and were allowed to drink and eat freely. The broilers were randomly divided into six groups with three replicates cages of six broilers each. The following groups were used: blank control group (B), cyclophosphamide model group (M), ASPS-PD control group (P), ASPS-PD low dose group (L) and high dose group (H) and *Astragalus* polysaccharide group (ARPS, PC). The experimental design is shown in Table 1 and the experiment lasted for 24 days. After 3 days of acclimatization, B and P groups were administered by intraperitoneal injection with 0.3 ml saline and H, L, PC and M groups were injected with 80 mg/kg CY at 4–6 day. During 4–24 day, P and H groups was gavaged with 80 mg/kg ASPS-PD, L group was gavaged with 40 mg/kg ASPS-PD, PC group was gavaged with 40 mg/kg ARPS. B and M groups was gavaged saline solution as control. The diets and nutrient levels in accordance with National Research Council requirements (Table 2).

**Table 1:** Animals experimental design.

**Days**	**B**	**P**	**H**	**L**	**PC**	**M**
0–3 d	Adaptive feeding					
4–6 d	Saline			80 mg/kg CY		
4–24 d	Saline	80 mg/kg ASPS-PD		40 mg/kg ASPS-PD	40 mg/kg ARPS	Saline

**Table 2:** Base diet composition and nutrient levels.

**Ingredients**	**Content/%**	**Nutritional level**	**Content/%**
Maize	58.37	Crude protein≥	20.00
Wheat flour	13.35	Moisture ≤	13.00
Soya bean meal	21.75	Crude fiber ≤	4.50
Soya bean oil	2.06	Crude ash ≤	7.00
Stone powder	1.55	Calcium	0–0.80
Calcium hydrogen phosphate	1.70	Total phosphorus	0.60
Amino acid	0.51	Sodium chloride	0.30–0.80
Trace element premix	0.71	Methionine	0.50–0.90

### 2.4 Measurement indicators and methods

#### 2.4.1 Growth performance

During the trial period, feed intake were recorded in detail for each group. Broilers were weighed every 7 days (fasting for 2 h before conducting), and average daily gain (ADG), average daily feed intake (ADFI), feed conversion ratio (FCR) were calculated.


$$\text{FCR=}\frac{\text{Total feed intake}}{\text{Total weight gain}}$$

#### 2.4.2 Determination of immune organ index

On days 7, 14, and 21, six broilers were randomly selected and weighed. The spleen, thymus, and bursa were removed after euthanasia and accurately weighing for calculation of the immune organ index.


$$\text{Immune organ index }(\text{mg/g})\text{=}\frac{\text{ organ mass}}{\text{living body mass}}$$

#### 2.4.3 Determination of cytokines and immunoglobulins

On days 7, 14, and 21, six broilers were randomly selected, the serum samples were separated, and frozen at −20°C. Cytokines and immunoglobulins were determined by enzyme-linked immunosorbent assay (ELISA).

#### 2.4.4 HE staining

Histopathological sections of immune organs were prepared and stained with haematoxylin and eosin, the morphological and pathological changes was observe under the microscope (28).

### 2.5 Gut microbiota analysis

#### 2.5.1 DNA extraction and PCR amplification

By the standard fecal sampling procedure and strict adherence to the principle of aseptic operation, cecal feces were collected from broilers on day 24 of the experiment and stored frozen at −80°C. The CTAB method was used to extract total DNA from pooled fecal matter. The purity and integrity of the whole DNA was checked on a 1% agarose gel. Primers 341F (5′-CCTAYGGRBGCASCAG-3′) and 806R (5′-GGACTACNNGGGTATCTAAT-3′) were used to amplify the V3-V4 highly variable region (31, 32) of the bacterial 16S rRNA gene, and amplicons from each PCR sample were normalized to an equimolar quantity (33) (New England Biolabs), 2 μM forward and reverse primers and ~10 ng template DNA (34). PCR products were mixed equally. Universal DNA (TianGen, China) was used for purification.

#### 2.5.2 Library generation and Illumina sequencing

Libraries were prepared using the NEB Next^®^ Ultra DNA Library Prep Kit (35). The built libraries were detected and quantified by Q-PCR using an Agilent 5400; After the libraries were qualified, they were sequenced using the Illumina sequencing platform.

#### 2.5.3 Bioinformatics analysis

The final feature list and feature sequences were obtained after quality control, trimming, denoising, splicing, and chimera removal using the DADA2 method recommended by QIIME2 (36). Alpha and beta diversity were calculated by randomly normalizing to the same sequence using QIIME2 (37–39).

### 2.6 Data analysis

Data were analyzed for statistical purposes using SPSS 21 software for multiple analysis and significance. Graphing was performed using OriginPro 2021 software. Gut microbiota diversity data were analyzed using the Micromeritics Alliance Bioscience Cloud website (https://www.bioincloud.tech).

## 3 Results

### 3.1 Effects on growth performance of broiler

The broilers in the B and P groups were in good spirits. The broilers in the M group were depressed, preferred to hug, and did not move well. As shown in Table 3, the body weight and ADG of the M group were significantly lower than those of the B and P groups (*P* < 0.05), and the FCR of the M group was much higher than those of B and P groups (*P* < 0.05), indicating the success of the cyclophosphamide immunodeficiency model. The final weight and ADG of group M were significantly lower than those of groups L, H, and PC, and the body weight of group L was considerably lower than that of group B, and there was no significant difference between group H and PC; the FCR of group M was higher than that of groups L, H and PC, and there was no significant difference between group PC and H. This also indicated a difference between the group M and group PC, as well as group M and group PC. The suggesting that ASPS-PD had some regulatory effect on the cyclophosphamide induced broiler immunodeficiency.

**Table 3:** Effect of ASPS-PD on production performance of broiler.

**Groups**	**Production performance**					
	**B**	**M**	**P**	**H**	**L**	**PC**
Initial weight (g)	71.96 ± 1.63	72.40 ± 1.78	71.89 ± 1.61	72.37 ± 1.89	72.10 ± 0.96	72.27 ± 1.42
Final weight (g)	276.76 ± 7.67	215.71 ± 9.45	293.40 ± 7.60	268.48± 10.39	256.67 ± 13.37	263.74 ± 26.73
ADG (g)	9.75 ± 0.39^c^	6.82 ± 0.39^a^	10.54 ± 0.33^d^	9.33 ± 0.46^c^	8.77 ± 0.59^b^	9.46 ± 0.63^c^
ADFI (g)	57.26	55.09	56.94	58.93	57.24	58.13
FCR	5.87	8.08	5.40	6.31	6.52	6.14

Different letters on the columns represent significant differences (*P* < 0.05).

### 3.2 Effects on immune organ index in broilers

#### 3.2.1 Effects on thymus index in broilers

Table 4 shows that on 7, 14, and 21 days, the thymus index of all groups showed some growth, with the M group showing the lowest growth. On 7, 14, and 21 d, group M was significantly lower than group B, indicating that cyclophosphamide negatively affected thymus growth in broilers. On 7 d, there was no significant difference between the thymus index of group B and group P. on 14 and 21 d, there was a substantial difference between the thymus index of groups B and P, and the thymus index of group P were higher than group B, indicating that ASPS-PD could improve the thymus index of broilers. On 21 d, there was a significant difference between groups H and PC, and the difference was higher than that of group PC. This suggests that ASPS-PD has a regulatory effect on cyclophosphamide-induced thymic damage in broilers.

**Table 4:** Effect on thymus index of broiler.

**Groups**	**Thymus index (mg/g)**		
	**7 d**	**14 d**	**21 d**
B	3.80 ± 0.046^c^	4.56 ± 0.050^c^	5.21 ± 0.071^b^
M	2.81 ± 0.030^a^	3.35 ± 0.086^a^	4.55 ± 0.067^a^
P	3.61 ± 0.751^c^	5.66 ± 0.041^d^	6.49 ± 0.070^f^
H	3.11 ± 0.125^b^	4.37 ± 0.189^bc^	6.05 ± 0.171^e^
L	3.06 ± 0.082^b^	4.27 ± 0.116^b^	5.45 ± 0.070^c^
PC	2.68 ± 0.421^a^	4.41 ± 0.702^b^	5.77 ± 0.092^d^

Different letters on the columns represent significant differences (*P* < 0.05).

#### 3.2.2 Effects on bursa index in broilers

Table 5 shows that on 7, 14, and 21 days, the bursa index increased to some extent in all groups, with the lowest increase in the M group. At 7, 14, and 21 d, the bursa index of group M was significantly lower than that of group B, indicating that cyclophosphamide affects the growth of the bursa in broilers. The bursa index of group B was significantly higher than that of group P on 7 d, and there was no significant difference between group B and group P at 14 and 21 d, indicating that ASPS-PD had the ability to enhance bursa index in broilers. There was no significant difference between group M and H, L and PC compared to group M at 7 d and 14 d, and at 21 d there was no significant difference between group H and PC compared to group H and PC, which was significantly higher than that of group M. The results of the study showed that group B was significantly higher than group P at 7 d. The results showed that cyclophosphamide can impair broiler immunity. This may indicate that cyclophosphamide causes atrophy of the bursa in broiler, and ASPS-PD has a regulatory effect on cyclophosphamide-induced atrophy of the bursa in broiler.

**Table 5:** Effect on bursa index of broiler.

**Groups**	**Bursa index (mg/g)**		
	**7 d**	**14 d**	**21 d**
B	2.78 ± 0.042^d^	2.89 ± 0.045^b^	4.19 ± 0.095^c^
M	1.06 ± 0.055^ab^	1.52 ± 0.047^a^	0.85 ± 0.071^a^
P	2.11 ± 0.095^c^	2.99 ± 0.050^b^	4.43 ± 0.055^c^
H	1.16 ± 0.100^b^	1.58 ± 0.055^a^	1.76 ± 0.030^b^
L	1.01 ± 0.026^a^	1.54 ± 0.056^a^	0.97 ± 0.050^a^
PC	1.13 ± 0.060^ab^	1.49 ± 0.029^a^	1.61 ± 0.38^b^

Different letters on the columns represent significant differences (*P* < 0.05).

#### 3.2.3 Effects on spleen index in broilers

From Table 6, it can be seen that from 7 to 14 d, the spleen index of M, H, L, and PC groups decreased, and on 21 d, the spleen index of all groups except the M group showed different increases. The difference between the H, L, and PC groups and the B group was insignificant. From 7 to 21 d, the spleen index of the B group and P group did not change much, but the P group was significantly higher than the B group. It indicated that cyclophosphamide lead to splenomegaly and ASPS-PD had a therapeutic effect on cyclophosphamide-induced splenomegaly in broilers.

**Table 6:** Effects on spleen index in broiler.

**Groups**	**Spleen index (mg/g)**		
	**7 d**	**14 d**	**21 d**
B	1.13 ± 0.050^b^	1.28 ± 0.085^c^	1.13 ± 0.051^b^
M	0.93 ± 0.051^a^	0.87 ± 0.055^ab^	0.788 ± 0.065^a^
P	1.41 ± 0.065^c^	1.33 ± 0.065^c^	1.37 ± 0.070^c^
H	1.12 ± 0.056^b^	0.86 ± 0.055^ab^	1.08 ± 0.065^b^
L	1.09 ± 0.055^b^	0.82 ± 0.070^a^	1.03 ± 0.055^b^
PC	1.06 ± 0.055^b^	0.99 ± 0.055^b^	1.07 ± 0.158^b^

Different letters on the columns represent significant differences (*P* < 0.05).

### 3.3 Effects on cytokines in broilers

#### 3.3.1 Effects on IL-2 serum levels in broilers

As can be seen from Table 7, on 7, 14, and 21 d, IL-2 showed an increasing trend in groups B and P; the remaining four groups were significantly higher on 14 d but decreased on 21 d. On 21 d, group H tended to be closer to group B, significantly higher than the model group and insignificantly different from the PC group.

**Table 7:** Effect on serum IL-2 levels in broilers.

**Groups**	**IL-2 (**μ**g/mL)**		
	**7 d**	**14 d**	**21 d**
B	118.97 ± 1.27^b^	137.94 ± 1.61^a^	142.54 ± 0.78^b^
M	98.38 ± 0.60^a^	168.32 ± 5.42^c^	115.41 ± 2.34^a^
P	117.49 ± 4.29^b^	138.16 ± 2.93^a^	149.38 ± 3.62^b^
H	119.61 ± 2.25^b^	150.06 ± 4.09^ab^	137.32 ± 4.28^b^
L	119.05 ± 3.66^b^	158.08 ± 3.49^bc^	126.37 ± 4.72^a^
PC	113.58 ± 3.55^b^	149.54 ± 6.26^ab^	141.59 ± 4.08^b^

Different letters on the columns represent significant differences (*P* < 0.05).

#### 3.3.2 Effects on serum IFN-γ levels in broilers

As shown in Table 8, the M group was significantly lower than group B from 7 to 21 d. Group B and Group P were insignificant on 7 d, and Group B was significantly lower than Group P on 14 and 21 d, indicating that ASPS-PD had a positive correlation with IFN-γ. At 7 d, groups M, H, L, and PC were significantly lower than groups B and P, suggesting that cyclophosphamide had the effect of lowering IFN-γ secretion. Group H was considerably higher than Group M and was insignificant with Group B on 21 d. Group H was significantly higher than M, H, L, and PC groups were considerably lower than B and P groups on 7 d, indicating that cyclophosphamide has the effect of reducing IFN-γ secretion.

**Table 8:** Effect on serum IFN-γ levels in broilers.

**Groups**	**IFN-**γ **(**μ**g/mL)**		
	**7 d**	**14 d**	**21 d**
B	115.30 ± 0.52^c^	118.126 ± 4.66^c^	110.56 ± 1.99^ac^
M	81.79 ± 1.26^a^	90.55 ± 1.30^a^	93.96 ± 1.96^a^
P	117.94 ± 1.75^d^	130.06 ± 3.75^d^	124.08 ± 4.45^d^
H	85.83 ± 3.24^b^	108.78 ± 4.47^ac^	116.11 ± 1.24^cd^
L	82.17 ± 0.29^a^	103.22 ± 0.95^b^	103.91 ± 4.61^b^
PC	83.22 ± 0.21^a^	110.54 ± 5.15^ac^	109.12 ± 3.04^ac^

Different letters on the columns represent significant differences (*P* < 0.05).

#### 3.3.3 Effects on serum IgG1 levels in broilers

As shown in Table 9, the IgG1 of all groups increased to a certain extent from 7 to 21 d, and the increase was the least in group M. Group M was significantly lower than group B on 7 d. Groups H, L, and PC were insignificant compared with group B on 21 d but were considerably higher than group M. Group B was significantly lower than Group P. This indicates that ASPA-PD can promote the secretion of IgG1 and improve the effect of cyclophosphamide in reducing IgG1 secretion. This suggests that ASPA-PD can stimulate the secretion of IgG1 and enhance the effect of cyclophosphamide in reducing the secretion of IgG1.

**Table 9:** Effects on serum IgG1 levels in broilers.

**Groups**	**IgG1 (**μ**g/mL)**		
	**7 d**	**14 d**	**21 d**
B	118.59 ± 4.39^b^	128.45 ± 7.91^abc^	128.55 ± 4.57^ab^
M	104.57 ± 2.93^a^	109.54 ± 6.68^a^	117.14 ± 2.27^a^
P	124.78 ± 1.80^b^	149.63 ± 6.06^c^	156.77 ± 5.63^c^
H	123.69 ± 4.73^b^	137.77 ± 5.52^bc^	132.22 ± 5.61^b^
L	118.39 ± 3.80^b^	123.47 ± 2.94^ab^	129.03 ± 1.98^b^
PC	121.49 ± 5.04^b^	134.92 ± 5.02^bc^	134.06 ± 3.88^b^

Different letters on the columns represent significant differences (*P* < 0.05).

### 3.4 Effects on the morphology of broiler immune organs

#### 3.4.1 Effects on the histomorphology of the bursa of broilers

**Figure 1:**
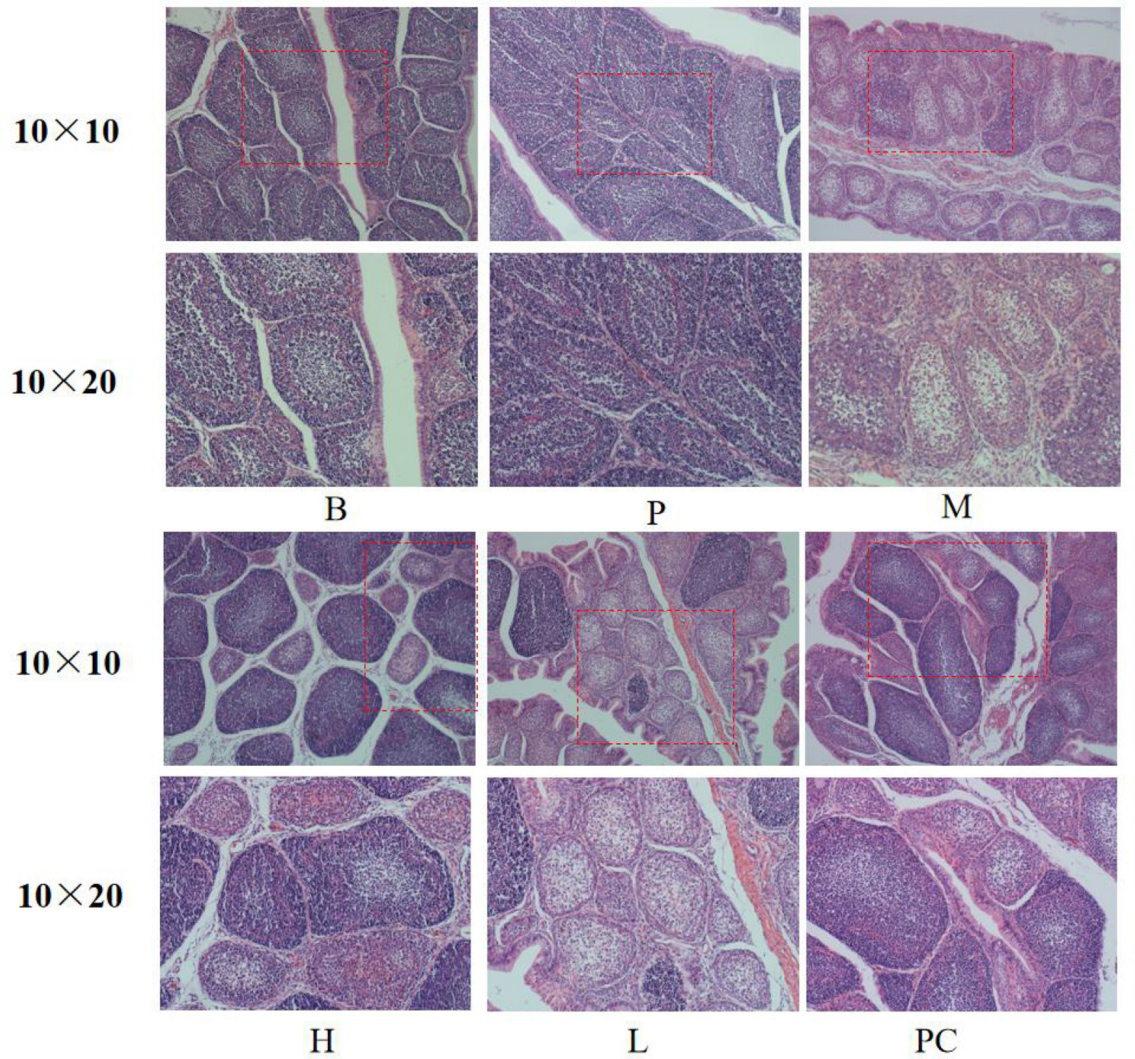
Effect on histomorphology of bursa of broiler.

As shown in Figure 1, the cortical and medullary boundaries of groups B and P were clear, the lymphocytes were arranged orderly, and the lymphocytes in group P were significantly more than those in group B. The cortical and medullary boundaries of group M became less precise, the number of lymphocytes decreased significantly, and the follicles showed atrophy. The cortical and medullary borders of group M became less and less obvious, the number of lymphocytes decreased significantly, and the follicles showed atrophy. The cortical and medullary borders of group L and group H were gradually enhanced, the number of lymphocytes gradually increased, and the area of the follicles was slightly smaller. Still, there was no significant difference in the number of lymphocytes between groups H and PC. Compared with the PC group, the follicular area of the H group was slightly smaller, but there was no noticeable difference in the number of lymphocytes.

#### 3.4.2 Effect on thymic histomorphology in broilers

**Figure 2:**
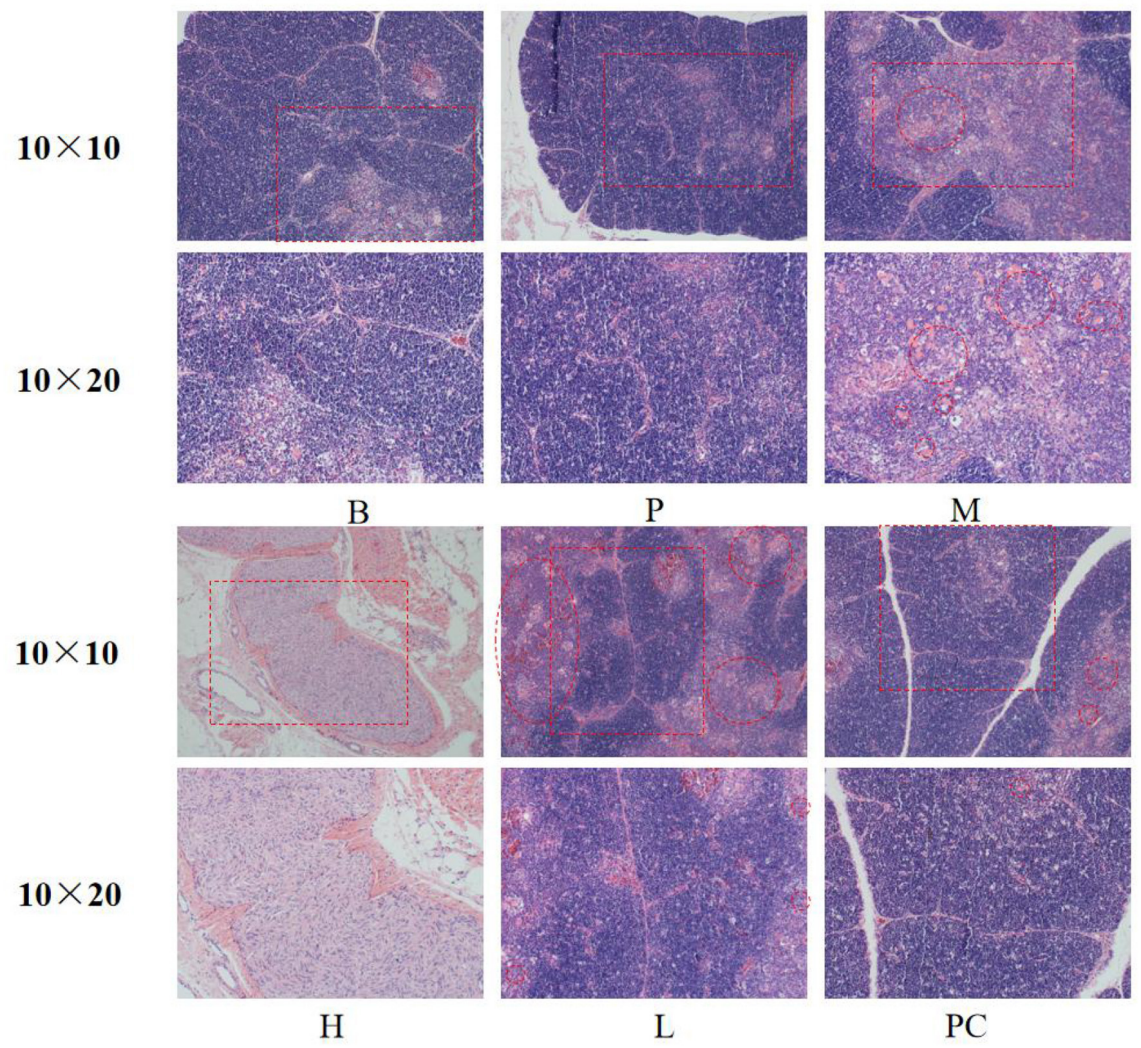
Effect on the morphology of thymus tissue.

As shown in Figure 2, the thymus structure was standard in Groups B and P. Group M caused congestion in the medullary region of the thymic lobules and a decrease in cortical and medullary lymphocytes. Thymic cortical and medullary lymphocytes were increased compared to Group B, and mild congestion was observed in Groups H, L, and PC.

#### 3.4.3 Effects on the spleen histomorphology of broiler

**Figure 3:**
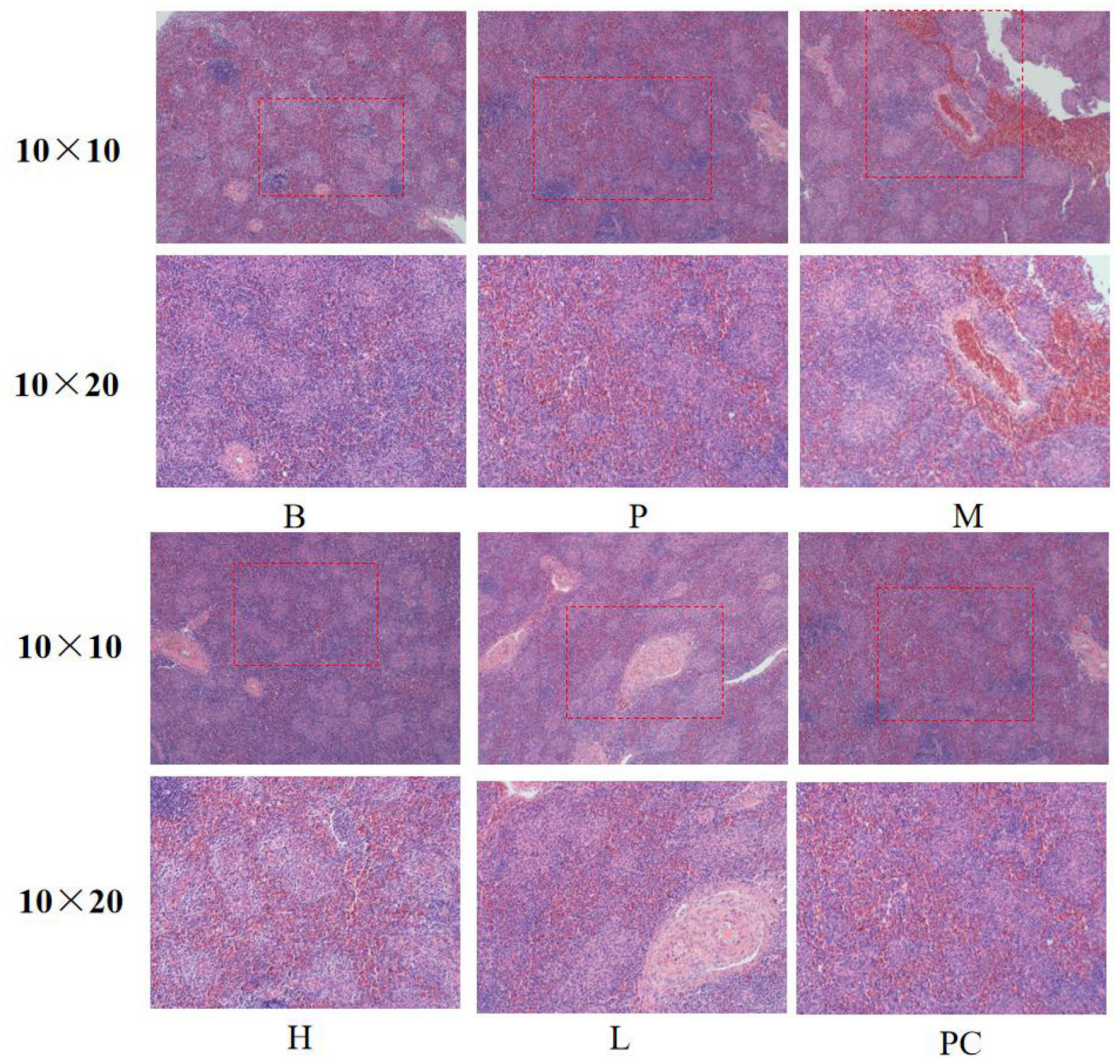
Effect on the spleen histomorphology of broiler.

As shown in Figure 3, the spleens of Group B and Group P had standard structure; the lymphocytes in the splenic nodes were closely arranged, clear in outline, and more numerous; the white and red medulla were evenly distributed; the lymphocytes and splenic nodes of group M and group L were significantly less than those of the B group, and the arrangement of lymphocytes was sparse; the lymphocytes in the splenic nodes of group H and group PC were more closely arranged and clearer in outline than those of group M. The lymphocytes in group H and PC's splenic nodes were more densely arranged and more apparent in shape than those in group M.

### 3.5 Analysis of gut microbiota

#### 3.5.1 OTU analysis

**Figure 4:**
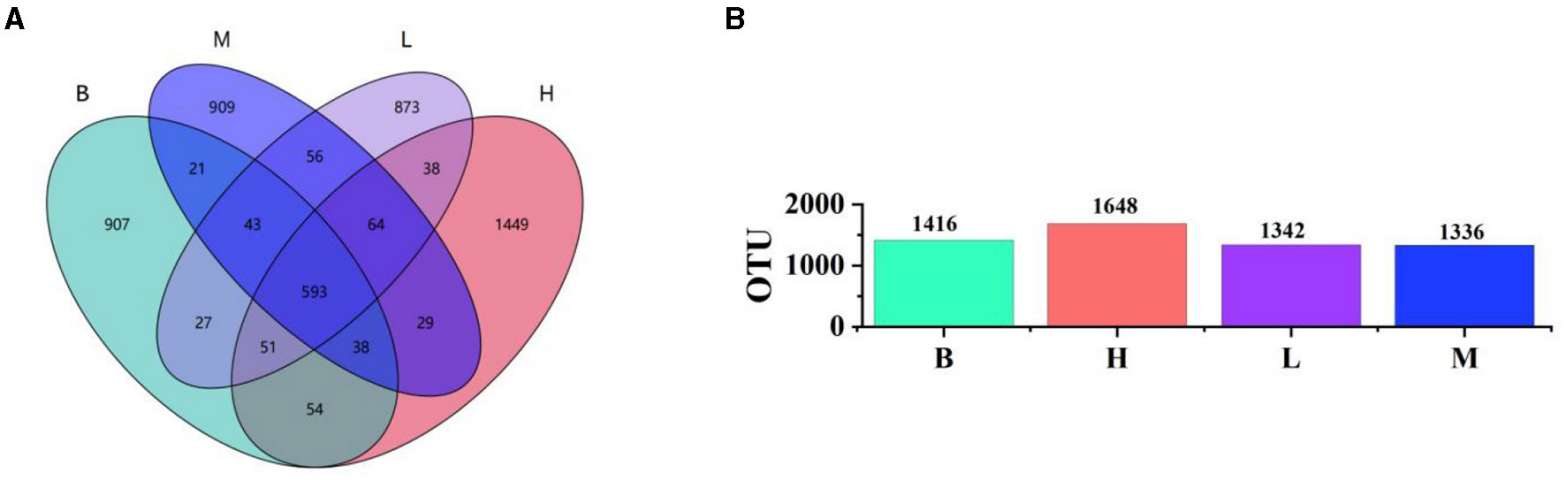
Analysis of the four experimental groups at the OTU level. **(A)** Wayne's plot; **(B)** bar chart of OTU species in each group.

We collected and sequenced 16S rRNA from the cecal contents of the groups B, M, H, and L. The 2,710,845 sequences were obtained. The average number of sequences generated from each broiler cecum sample was 88,380.5, and all sequences were classified into operational taxonomic units (OTUs) with a similarity level of 97%, which were classified into 24 species, 52 orders, 77 phyla, 127 families, and 151 genus. The number of unique and shared species in the cecum of broilers in different groups is clearly shown in the Wayne diagram (Figure 4A). The number of shared OTU species is 498, among which 613 OTUs are unique to group M and 603 shared with group B, 653 shared with group H and group B, and 639 shared with group L and the model group, and the number of shared OTUs with group L is 639. The results of the OTU species show that groups H and L are closer to group B than group M, indicating that ASPS-PD regulates the normalization of the gut flora. As can be seen from the bar graph B (Figure 4B), the abundance of the flora in all groups was higher than that in group M.

#### 3.5.2 Alpha diversity analysis

**Figure 5:**
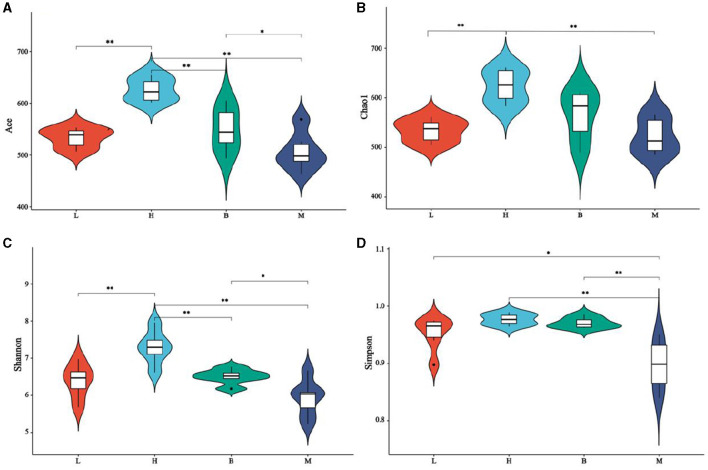
Alpha diversity analysis. (**A)** Ace index; **(B)** Chao index; **(C)** Shannon index; **(D)** Simpson index.

Alpha diversity is a comprehensive index that reflects the richness and evenness of the gut flora. The Chao and Ace index were mainly used to evaluate the richness of the flora in each group; the Shannon index and Simpson diversity index were used to highlight the diversity among the flora in each group. In addition, good coverage indices were analyzed, all of which were >0.99, indicating the credibility of the data. As shown in Figure 5, for Chao (b), group H was higher than groups L and M (*P* < 0.01) and not significant with group B. For the Ace (a) index, group H scored much higher than groups L, M, and B (*P* < 0.01), and group B scored much higher than group M (*P* < 0.05) but was not meaningful with group L. The results of the Chao and Ace indices show that group H has the highest population richness, followed by group B, which is consistent with the OUT index. Group H is significantly larger than groups L, M, and B on the Shannon (c) index (*P* < 0.01), and group B is more significant than group M (*P* < 0.05) but not statistically different with group L. Groups H and B on Simpson (d) are significantly larger than group M (*p* < 0.01), the B group is considerably larger than group L (*P* < 0.05), and the L group is significantly larger than group M (*P* < 0.05). The Shannon and Simpson indices results showed that groups B and H populations were more evenly distributed. Alpha diversity analysis indicated that cyclophosphamide caused a decrease in the diversity and homogeneity of cecal flora in broilers, and the use of ASPS-PD could positively mitigate the adverse effects of cyclophosphamide.

#### 3.5.3 Beta diversity analysis

**Figure 6:**
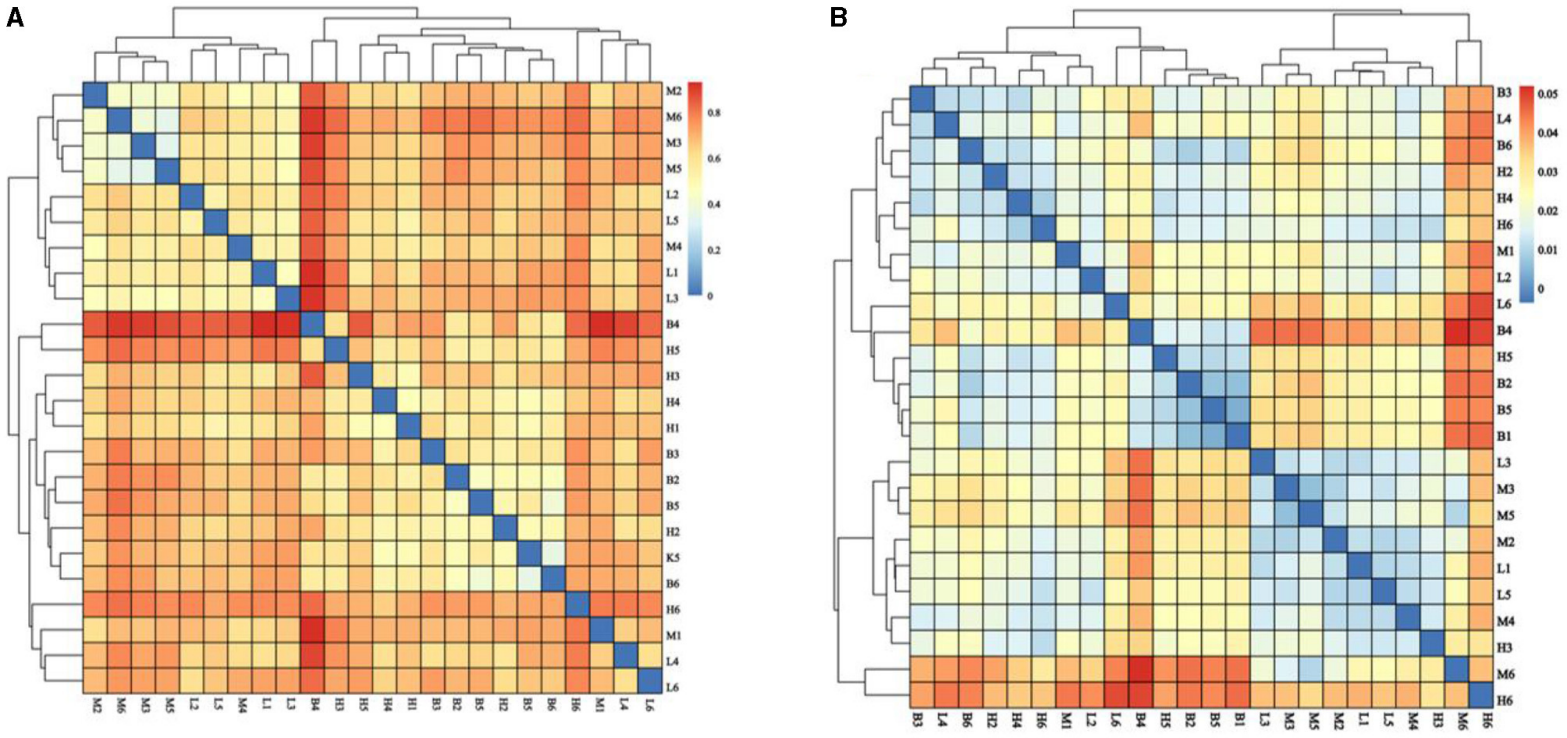
Analysis of the β-diversity index. **(A)** Bray Curtis; **(B)** Weighted Unifrac.

**Figure 7:**
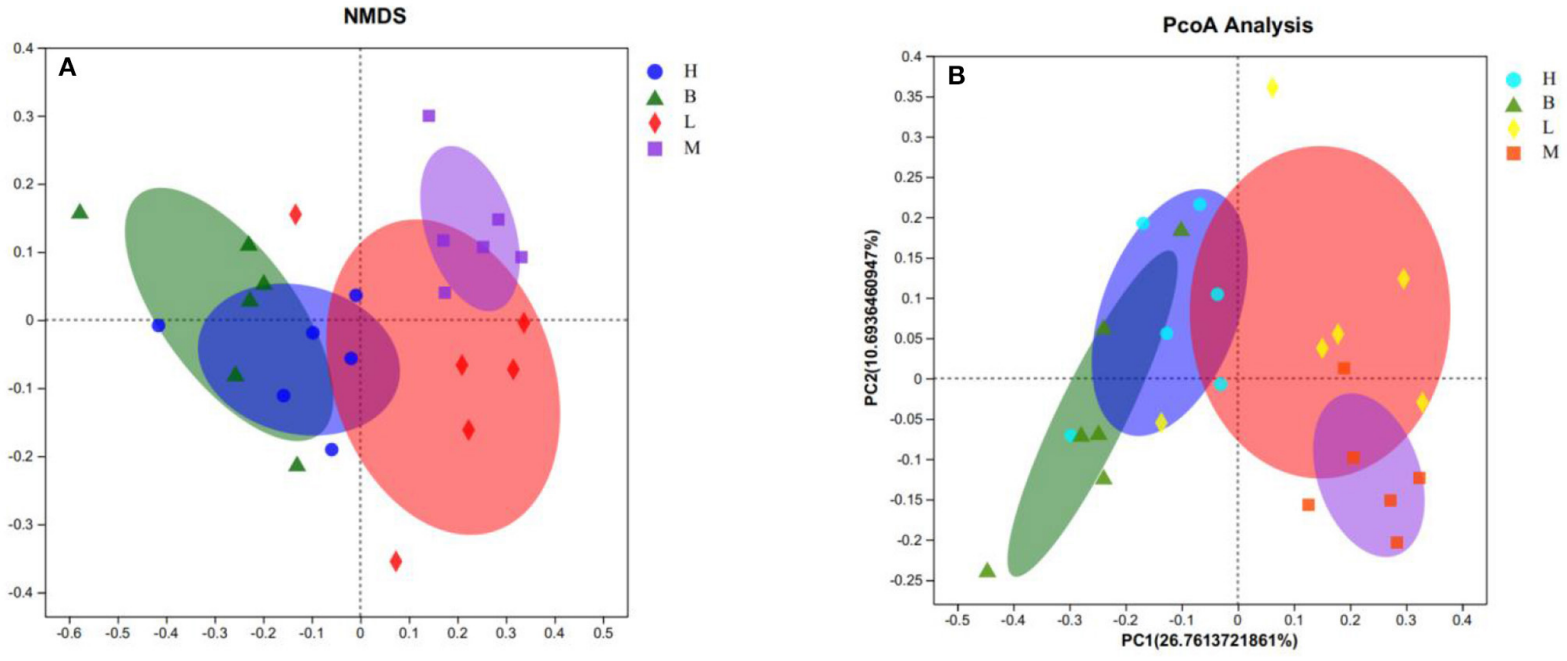
NMDS and PCOA analyses. **(A)** NMDS score plot; **(B)** PCOA analysis plot.

Beta diversity analysis explores similarities or differences between groups of samples by analyzing samples between groups. As shown in Figure 6, with the largest difference between groups M and B, the smallest difference with group L, and the smallest difference between group B and H. The further analyses, NMDS and PCOA, were used to analyse the gap between the groups more clearly. As shown in Figure 7A, in NMDS group B is mainly located in the second and third quadrants, group M in the first quadrant, group H in the third and fourth quadrants and group L in the first and fourth quadrants. As shown in Figure 7B, group B in PCOA is mainly in the second and third quadrants, group M is in the fourth quadrant, group H is in the third quadrant and group L is in the first quadrant. The overlap of the confidence circles of group M and group B is almost 0 in both analyses, while the overlap of the confidence circles of group B with group H is the highest, and there is some overlap of the confidence circles of group L with those of groups M and H. From the analysis of the results, it can be seen that the difference between groups M and B was the largest and the difference between group H was the smallest, which means that ASPS-PD has a regulatory and restorative effect on the disruption of the intestinal flora of broilers caused by cyclophosphamide.

#### 3.5.4 Analysis of the composition of the intestinal microflora

##### 3.5.4.1 Analysis of microorganisms at the phylum level

**Figure 8:**
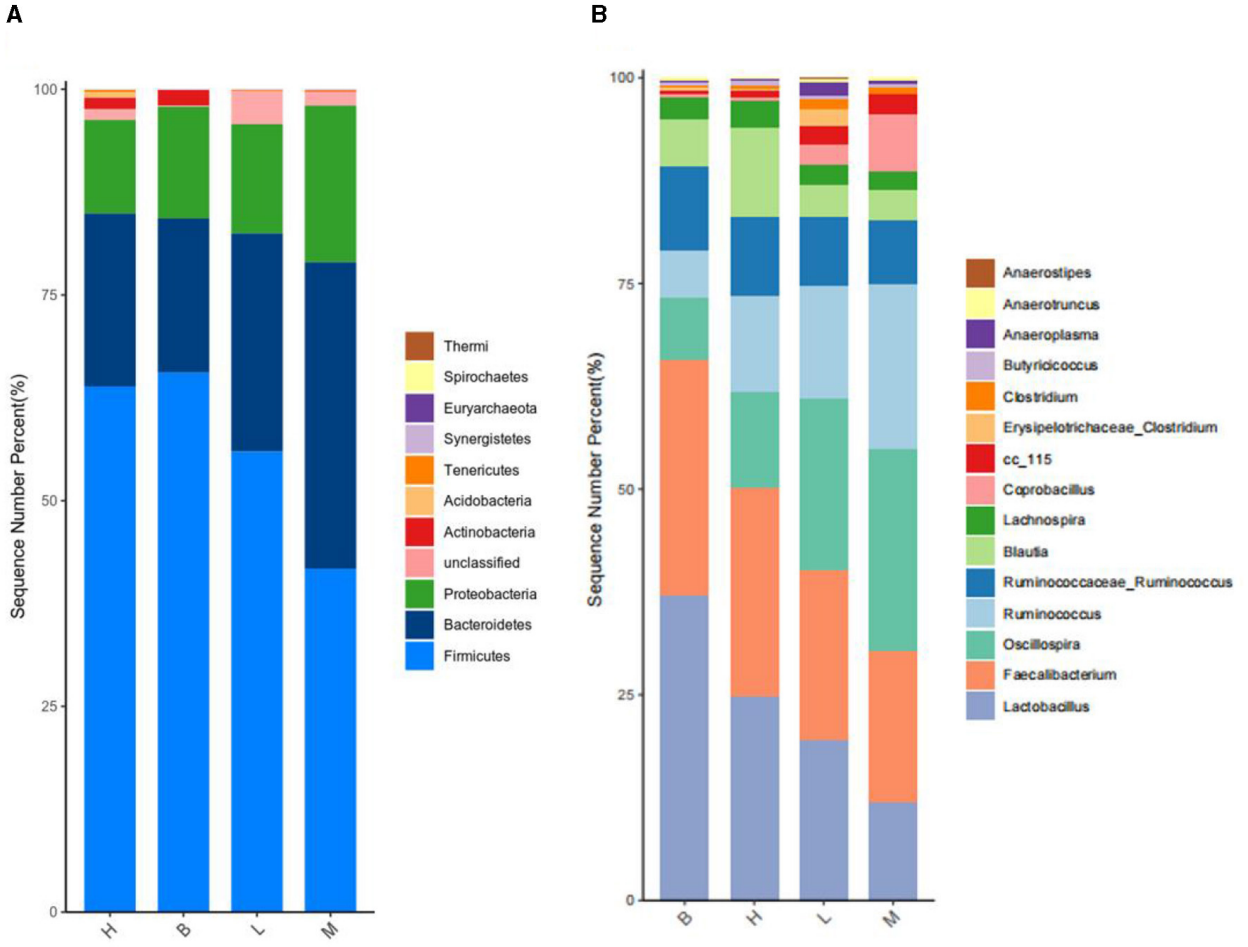
Species accumulation at phylum level and genus level. **(A)** Species accumulation at the phylum level; **(B)** species accumulation at the genus level.

A total of 24 species were identified based on taxonomic analysis, with the abundance of *Firmicutes* (41.72–65.56%) and the abundance of *Bacteroidetes* (18.74–38.26%) as the dominant species, followed by the abundance of *Proteobacteria* (11.37–19.02%). As shown by the cumulative plot of the first nine species at the phylum level (Figure 8A), the abundance of *Firmicutes* in group M was significantly reduced, and the abundance of *Proteobacteria* was increased relative to group B. However, there was a gradual increase in the abundance of *Firmicutes* and a gradual decrease in the abundance of *Proteobacteria* in groups L to H.

##### 3.5.4.2 Analysis of microorganisms at the genus level

A total of 151 genus were identified based on taxonomic analyses, with the abundance of *Lactobacillus, Faecalibacterium*, and *Oscillospira* as the dominant genus, and all three genus belonged to the Firmicutes. As shown in Figure 8B, a stacking diagram was constructed for the top 15 genus at the genus level, with the abundance of *Lactobacillus* and *Faecalibacterium* increasing from groups M, L, H, and B, and the abundance of *Oscillospira* decreasing.

#### 3.5.5 Specific colony analysis

**Figure 9:**
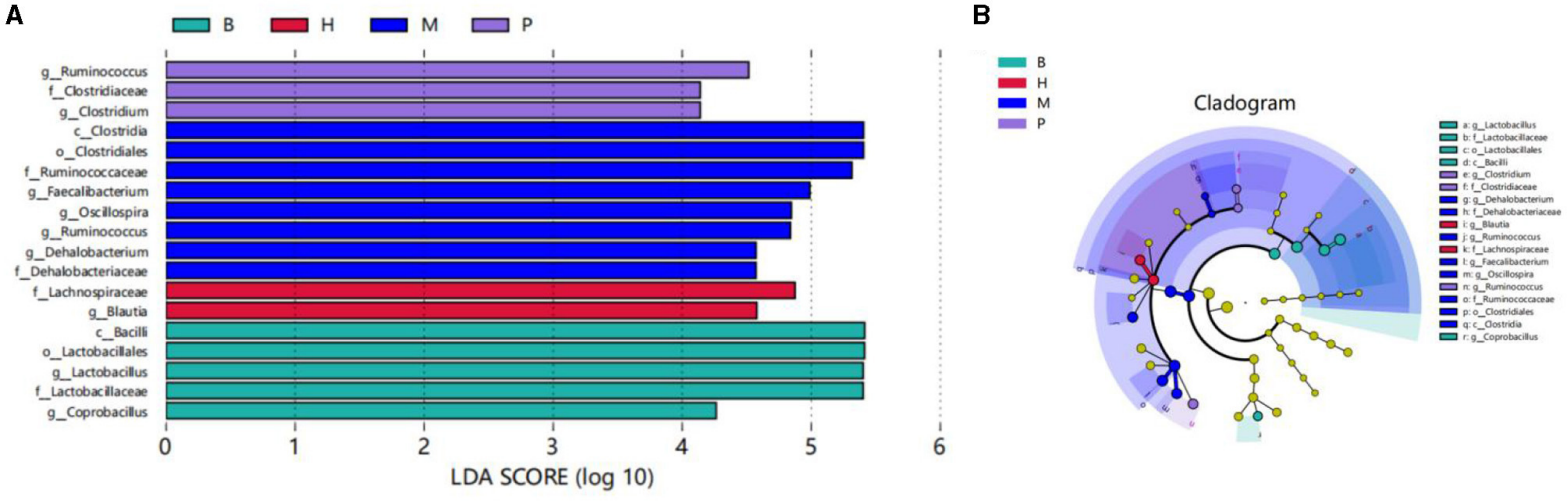
LEfSe analysis. **(A)** Distribution histogram based on LDA scores. LDA score (lg) > 4. **(B)** Cladogram.

The LEfSe method is based on a relative abundance table, a combination of non-parametric tests, and linear discriminant analysis. It is suitable for testing differences in the abundance of bacterial groups (40). The results showed that there were significant differences among the four groups with a total of 18 bacterial branches.The species with LDA scores greater than the basal set value (LDA > 4) are shown in the bar chart of LDA value distribution. As shown in Figure 9A, there were five different species in group B, two different species in group H, eight different species in group M, and three different species in group P. *Bacillus* and *Lactobacillus* scored highest in group B; *Lachnospiraceae* and *Bacill*i scored highest in group H; *Clostridiales* and *Clostridia* scored highest in group M; and *Ruminococcus* scored highest in group P. The highest scores were found in group B, group M. As shown in Figure 9B, the cladogram corresponds to the different taxonomic levels of phylum, order, family and genus from the inside to the outside, and the connecting lines between the levels represent the affinities. Each circle node represents a species, and a yellow node indicates that the difference between the subgroups is not significant, while a non-yellow node indicates that the species is a characteristic microorganism of the corresponding colored subgroup (with a significantly higher abundance in that subgroup). Colored sectors mark the subordinate taxonomic intervals of the characteristic microorganisms.

#### 3.5.6 Correlation analysis

**Figure 10:**
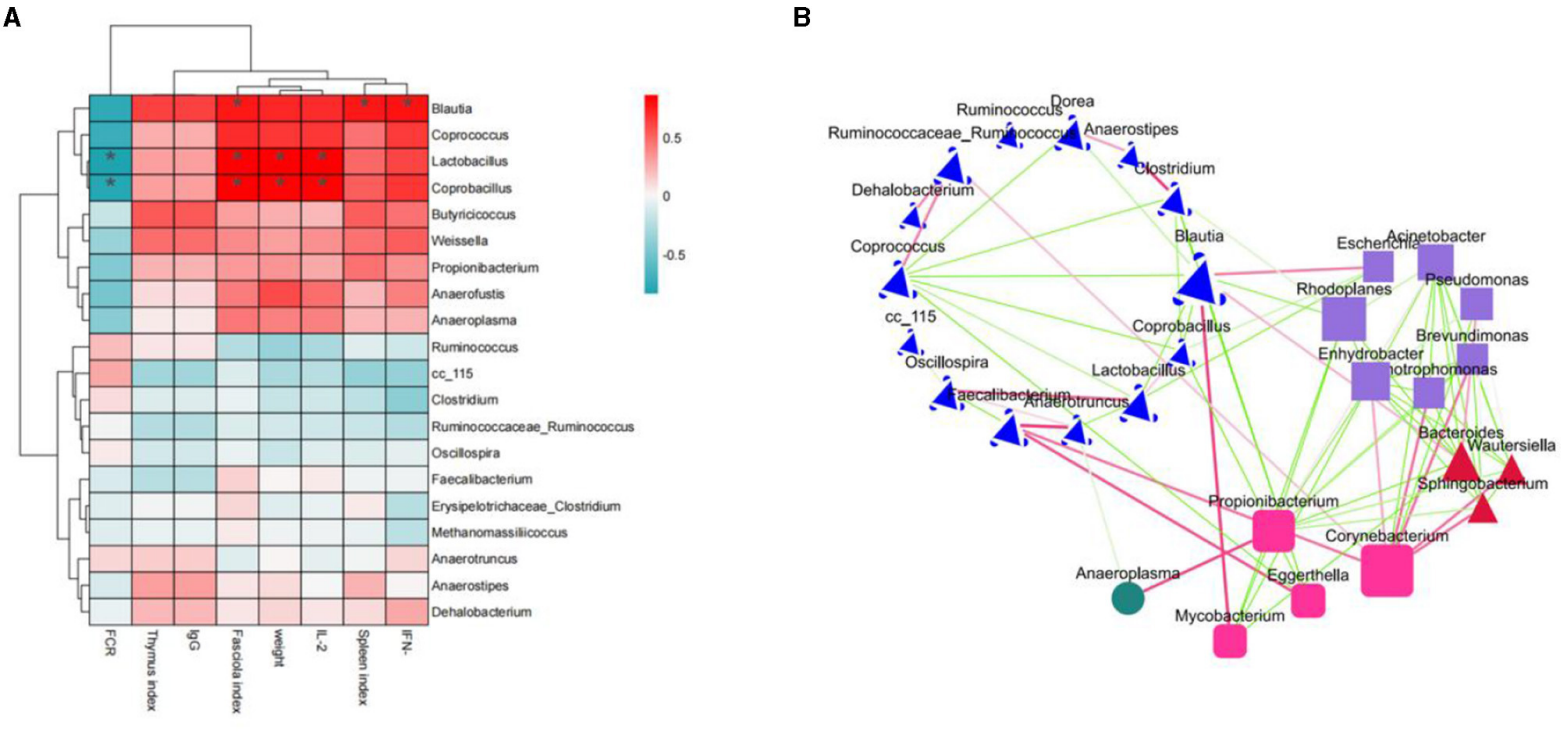
Correlation analysis. **(A)** Correlation heatmap; **(B)** species antagonistic/synergistic relationship map.

Correlation analyses were performed between the top 20 microorganisms at the genus level and immune factors, body weight, and organ index. As shown in Figure 10A, IFN-γ was significantly positively correlated with *Blautia*; spleen index was significantly positively correlated with *Blautia*; IL-2 was significantly positively correlated with *Enterococcus, Coprobacillus* and *Lactobacillus*; body weight and *Coprobacillus, Lactobacillus*; Bursa index and *Blautia, Coprobacillus, Lactobacillus*; FCR and *Coprobacillus* were significantly and negatively correlated. In the previous species analysis of microorganisms at the genus level, group H had the highest number of *Blautia*, and the number of *Lactobacillus* was increased compared to groups M and L. Therefore, the immunosuppression caused by cyclophosphamide modulation by ASPS-PD may be achieved by increasing the abundance of *Blautia* and *Lactobacillus*, which needs to be further explored in subsequent studies. In addition, the mutual antagonistic or synergistic effects between the individual microbial genus were analyzed at the genus level. In Figure 10B, two species are connected in red for synergistic relationships; the darker the color, the stronger the correlation, and in green for antagonistic relationships. Different shapes represent different phylum, e.g., the blue species belonging to the Firmicutes. This confirms that the dominant phylum in the previous analysis at the species level is the Firmicutes.

#### 3.5.7 KEGG analysis

**Figure 11:**
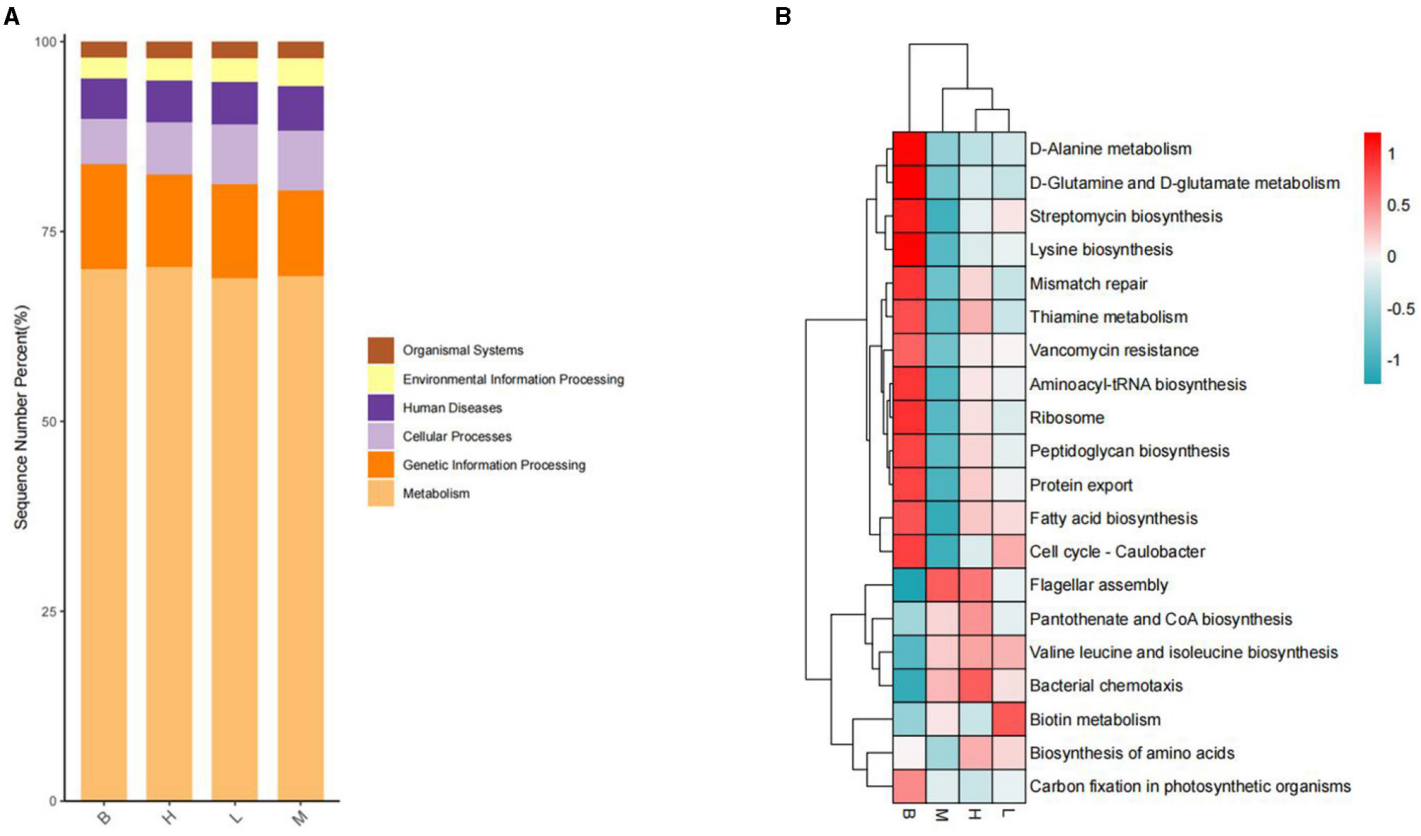
KEGG analysis. **(A)** L1 level stacking plot; **(B)** L3 level clustering plot.

**Figure 12:**
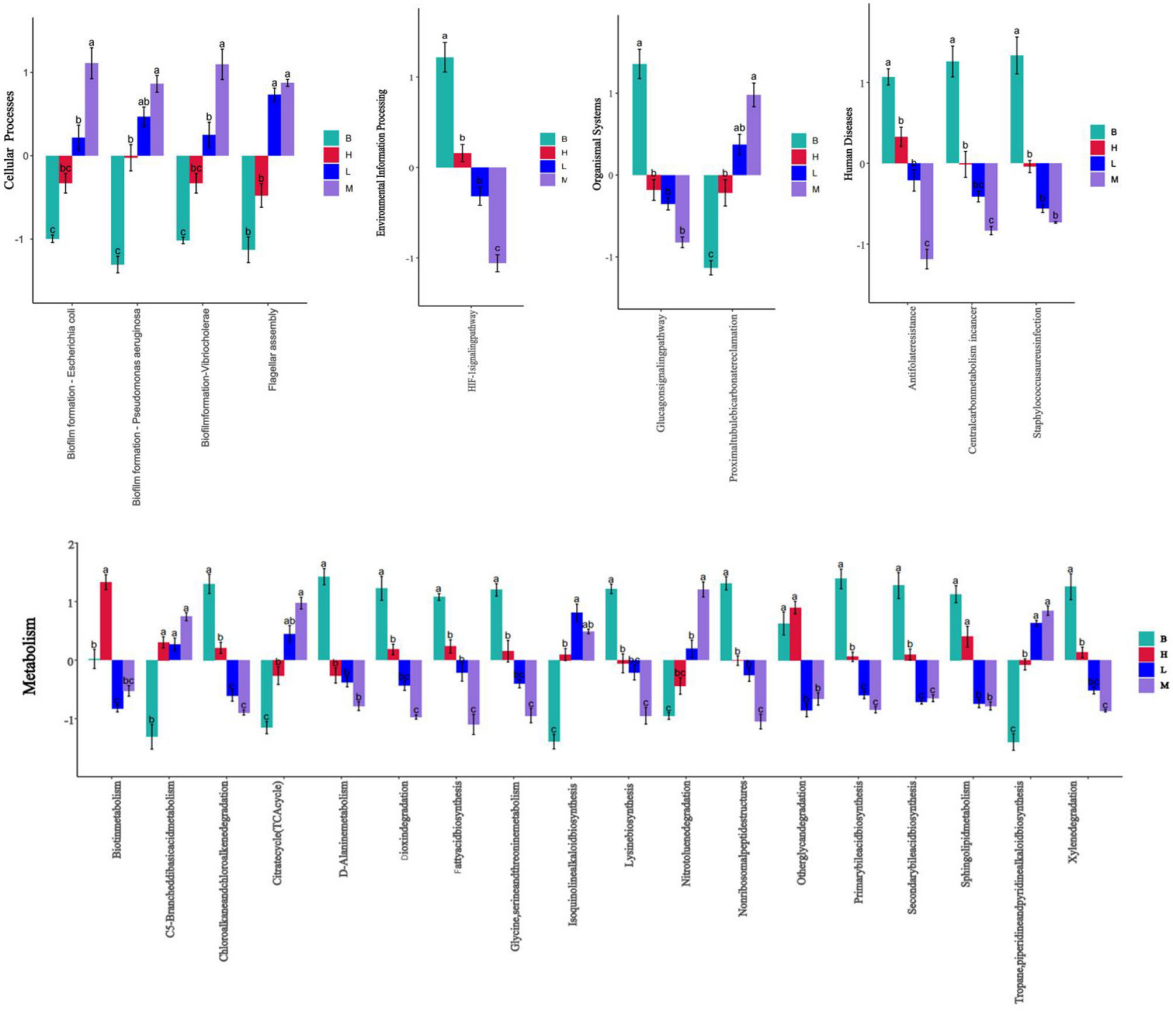
Significance of difference in KEGG at L3 level based on different scores at L1 level.

In the KEGG level functional abundance clustering plot, horizontal represents sample information, and vertical represents applicable annotation information. KEGG level 1 metabolic pathway analysis is shown in Figure 11; the Metabolism level is similar in four groups: genetic Information Processing has the most in group B and the least in group M; Cellular Processes have the least in group B, and the other groups have similar levels; Human Diseases and Organismal Systems 4 groups have identical levels; In Environmental Information Processing, group M has slightly higher levels. The analysis of KEGG level 3 is shown in Figure 11B, and combined with the significance of differences in Figure 12, in Cellular Processes, *E. coli* Biofilm formation—*Escherichia coli, Pseudomonas aeruginosa* Biofilm formation—*Pseudomonas aeruginosa, Vibrio cholera* Biofilm formation—*Vibrio cholerae* and other conditionally pathogenic bacteria were significantly higher in group M than group B. However, at the level of Metabolism Citrate cycle (TCA cycle), Fatty acid biosynthesis, Glycine, serine, and threonine metabolism, Primary bile acid biosynthesis in group B and secondary bile acid biosynthesis were significantly higher than those in group M. Glycine, serine, and threonine Glycine and serine both provide energy to the body during metabolism and the synthesis of threonine adenosine triphosphate, an essential hormone in cellular metabolism. Serine and threonine can also regulate the immune system and increase the body's immunity and antioxidant capacity.

## 4 Discussion

In modern breeding systems, broilers are often affected by various external factors such as disease, nutrition, and environment, resulting in growth disorders, poor health, and even high mortality due to poorly developed intestinal function and immature immunity (41). Polysaccharides are a group of powerful natural immunomodulation that can stimulate the growth of immune organs, stimulate immune cells, activate the complement-system and liberate cytokines (5, 42–45). Polysaccharides are used as green feed additives to enhance poultry immunity and disease resistance and regulate the balance of gut microorganisms. In this study, a model of cyclophosphamide immunosuppression in broilers was established to investigate the regulatory effects of ASPS-PD on the cyclophosphamide immunosuppression and its impact on gut microbiota in broilers.

Studies have shown that the growth performance of broilers in an immunosuppressed state is reduced, as evidenced by slower body weight growth and higher FCR (46, 47). In this experiment, the FCR increased, and body weight growth slowed down in group M compared to group B. Both groups H and L alleviated the effects caused by CY, and the ADG and FCR of broilers in group H and group B were not significantly different. Polysaccharides improve the growth performance of poultry, such as adding *Achyranthes bidentata* polysaccharides to the diet improved the body weight and average daily weight gain of broilers infected with *Escherichia coli* K88 (48). Shu et al. (49) showed that the polysaccharides of *Polygonatum sibiricum* had the effect of antagonizing CY-induced immunosuppression in chickens and improved the growth performance of chickens by increasing the DWG and decreasing the FCR, which is consistent with the present experimental results. Some studies have shown a strong correlation between the ability of polysaccharides to improve animal growth performance indicators and the amount added to the diet (50, 51). Wu et al. suggested after a 6-week feeding trial that the addition of 500, 1,000, and 2,000 mg/kg *Astragalus* polysaccharides (APS) to the diet was effective in increasing the weight gain of broilers, with the best results in the high-dose group (52). In present experiment, the high dose of ASPS-PD (80 mg/kg) was more effective in modulating the cyclophosphamide-induced decline in the growth performance of broilers.

Larger organ weights and indices indicate stronger humoral and cellular immunity (53, 54). In this experiment, by calculating immune organ indices on 7, 14, and 21 d and observing HE-stained sections of immune organs on 21 d, ASPS-PD could promote immune organ growth and reverse cyclophosphamide-induced damage to immune organs. The polysaccharides favored the increase of T lymphocyte percentage. They promoted the development of immune organs, as shown in the rise in the area of lymphoid follicles and the expansion of the white marrow area, suggesting that polysaccharide treatment promotes the formation of lymphocytes in the bursa (55). Studies have reported that *Astragalus* polysaccharide and γ-irradiated *Astragalus* polysaccharide supplementation increased the relative weights of the thymus and spleen and promoted the proliferation of T lymphocytes in broilers (46). *Enteromorpha* polysaccharides increased broiler bursa's relative weight (56) and improved bursa morphology, including inflammatory cell infiltration, cell structure disruption, and cell necrosis (57). *Camellia oleifera Cake* polysaccharides have also increased broilers' spleen and thymus weights and indices (58).

ASPS-PD was found to increase the immune factors in the broilers' serum, as measured by serum levels of IgG1, IFN-γ, and IL-2 on 7, 14, and 21 days. Polysaccharides extracted from plants can enhance the immunity of animal organisms by activating macrophages (59). Serum immunoglobulins (Ig), play an essential role in the immune system of poultry (21) and correlate with the state of immune function. Supplementation with LBP has been reported to increase serum concentrations of IgA, IgG, and IgM (60). Addition of AS polysaccharides (61) and *Astragalus* polysaccharides (52) to the diet enhances humoral immunity in broilers, resulting in increased serum levels of IgA and IgG. Wu et al. (62) reported that the addition of *inulin* produced more significant amounts of IL-6 and tended to increase the concentrations of IgA and IgM. This is consistent with the findings of Zhang et al. on the immunological effects of *Artemisia argyi* on broilers, where *Artemisia argyi* polysaccharides increased the expression levels of immune factors such as IFN-γ (63).

In this experiment, ASPS-PD improved the dysbiosis caused by CY through increasing the abundance of the Firmicutes and decreasing the abundance of the *Proteobacteria*. Many microorganisms in the gut are the first barrier against pathogens (61, 64). The distal gastrointestinal microbiota can ferment plant compounds to produce metabolites and further alter and reshape the gut microbial community through fermentation (65). Polysaccharides are a unique carbon source for gut bacteria and have beneficial microbial regulatory effects. This suggests that broilers treated with polysaccharides have a higher abundance of beneficial bacteria and a lower abundance of harmful bacteria (55). For example, supplementation with *oleaginous* tea cake polysaccharides promotes the growth of probiotic bacteria and can inhibit the population of pathogenic bacteria (58). *Astragalus* polysaccharide supplementation increased the abundance of Firmicutes and decreased the abundance of *Proteobacteria* (66). The increase in abundance of *Firmicutes* and the significant decrease in abundance of *Bacteroidetes* in this experiment, resulting in a reduction of the B/F ratio, was associated with weight gain (67, 68), suggesting that ASPS-PD has a beneficial effect on weight gain in broilers.

Treatment with polysaccharides increased the abundance of *Aspergillus, Anaplasma, Lactobacillus*, and *Ruminalococcus*. And it decreased the abundance of *Escherichia coli* and *Salmonella* (48, 55, 58, 61). *Inulin* supplementation has been reported to reduce the concentrations of *E. coli, Salmonella*, and *Campylobacter* in the cecum of broiler chickens and to increase *bifidobacteria* (69, 70). In this experiment, ASPS-PD was found to have a therapeutic effect on the cyclophosphamide-induced reduction of *F. prausnitzii* and *Lactobacillus*, a result also consistent with the findings of Long et al. (61). *F. prausnitzii* is an anaerobic Gram-negative bacterium, suggesting that feeding ASPS-PD may have a positive effect on the survival of *F. prausnitzii* survival by providing an anaerobic environment (71, 72). Stanley et al. showed that *F. prausnitzii* and *Ruminococcaceae* were significantly correlated with improved growth performance (73). Singh et al. also found that an increase in the *Firmicutes* was positively associated with a decrease in the F/G ratio (68). *F. prausnitzii* has been shown to produce butyric acid (74), which further improves intestinal integrity and absorptive capacity (75). Sokol et al. demonstrated that *F. prausnitzii* is a probiotic with anti-inflammatory properties, blocking NF-*k*B and IL-8 expression in Caco-2 cells (76). Therefore, *Clostridium pumilus* may further reduce gut inflammation and improve gut health. In addition, *Coprobacillus* was found to increase significantly in group M. Although the change was insignificant, there was also a decrease in this genus in group M compared to group B. *Lachnospira* has been shown to degrade plant fiber and produce short-chain fatty acids (77). The graph shows that *Blautia* is most abundant in group H compared to the other three groups. *Blautia* can use hydrogen and carbon dioxide to produce acetate. Acetate is a secondary source of competence for intestinal epithelial cells and a source of energy for muscle and brain tissue, inhibits pathogenic bacteria, and has anti-inflammatory properties.

In the LEfSe analyses, the differential species varied among the four groups. Correlation analyses were performed between the top 20 microorganisms at the genus level and immune factors, body weight, and organ indices. It was found that ASPS-PD could modulate immunity by increasing the abundance of *Blautia, Lactobacillus*, and *Coprobacillus*. Among them, FCR was significantly negatively correlated with *Lactobacillus* and *Coprobacillus*, which is in accordance with Stanley's study in 2016 (73). Stanley et al. (78) found that bacterial communities in the cecum that produce butyric acid and explicate cellulose and starch were negatively correlated with FCR, and this group of beneficial bacteria included *Lactobacillus, Ruminococcus*, etc.

In the L3 level analysis of KEGG, the cellular processes of biofilm formation of conditionally pathogenic bacteria such as *E. coli* and *Vibrio cholerae* were significantly higher in group M than in group B. However, B glycine and serine's metabolic levels were considerably higher than group M's. Glycine, serine, and threonine Glycine and serine both provide energy to the body during metabolism and the synthesis of threonine adenosine triphosphate, which is an essential hormone in cellular metabolism. Serine and threonine can also regulate the immune system and increase the body's immunity and antioxidant capacity.

## 5 Conclusion

In conclusion, ASPS-PD can restore growth performance, increase immune organ index and improve serum cytokine levels of IL-2 and IFN-γ and immunoglobulin IgG1 levels in CY-treated broilers. In addition, ASPS-PD can increase the diversity of microbial communities by increasing beneficial bacteria such as *Lactobacillus* and *Blautia* to modulate CY-induced intestinal flora dysregulation in broilers. These results suggest that ASPS-PD has the potential to ameliorate CY-induced immunosuppression as an effective immunomodulator or functional feed additive in broilers.

## Data availability statement

The data we uploaded to “NCBI” has been published under the accession number “PRJNA1108000” with the link “https://www.ncbi.nlm.nih.gov/sra/PRJNA1108000”.

## Ethics statement

The animal study was approved by Research Ethics Committee of Liaocheng University. The study was conducted in accordance with the local legislation and institutional requirements.

## Author contributions

JS: Funding acquisition, Resources, Supervision, Writing – review & editing. JX: Conceptualization, Data curation, Formal analysis, Investigation, Visualization, Writing – original draft. XW: Formal analysis, Investigation, Methodology, Writing – review & editing. RZ: Formal analysis, Investigation, Validation, Writing – review & editing. XZ: Investigation, Validation, Writing – review & editing. YY: Investigation, Validation, Writing – review & editing. XC: Funding acquisition, Resources, Supervision, Writing – review & editing.
